# Examining Additional Aspects of Muscle Function with a Digital Handgrip Dynamometer and Accelerometer in Older Adults: A Pilot Study

**DOI:** 10.3390/geriatrics5040086

**Published:** 2020-10-31

**Authors:** Sean Mahoney, Lukus Klawitter, Kyle J. Hackney, Lindsey Dahl, Stephen D. Herrmann, Bradley Edwards, Ryan McGrath

**Affiliations:** 1 Department of Health, Nutrition, and Exercise Sciences, North Dakota State University, Fargo, ND 58108, USA; sean.mahoney@ndsu.edu (S.M.); lukus.klawitter@ndsu.edu (L.K.); kyle.hackney@ndsu.edu (K.J.H.); 2 Sanford Health, Fargo, ND 58103, USA; lindsey.dahl@sanfordhealth.org; 3 Department of Geriatrics, Grand Forks, ND 58202, USA; 4 Sanford Research, Sioux Falls, SD 57104, USA; stephen.herrmann@profileplan.com; 5 Sanford Health, Sioux Falls, SD 57117, USA; bedward5@good-sam.com

**Keywords:** aging, geriatric assessment, muscle strength, muscle weakness, physical functional performance

## Abstract

Background: Maximal handgrip strength (HGS) could be an incomplete and unidimensional measure of muscle function. This pilot study sought to examine the relationships between maximal HGS, radial and ulnar digit grip strength, submaximal HGS force control, HGS fatigability, neuromuscular HGS steadiness, and HGS asymmetry in older adults. Methods: A digital handgrip dynamometer and accelerometer was used to collect several HGS measurements from 13 adults aged 70.9 ± 4.0 years: maximal strength, radial and ulnar digit grip strength, submaximal force control, fatigability, neuromuscular steadiness, and asymmetry. Pearson correlations determined the relationships between individual HGS measurements. A principal component analysis was used to derive a collection of new uncorrelated variables from the HGS measures we examined. Results: The individual HGS measurements were differentially correlated. Maximal strength (maximal HGS, radial digit strength, ulnar digits strength), contractile steadiness (maximal HGS steadiness, ulnar digit grip strength steadiness), and functional strength (submaximal HGS force control, HGS fatigability, HGS asymmetry, HGS fatigability steadiness) emerged as dimensions from the HGS measurements that we evaluated. Conclusion: Our findings suggest that these additional measures of muscle function may differ from maximal HGS alone. Continued research is warranted for improving how we assess muscle function with more modern technologies, including handgrip dynamometry and accelerometry.

## 1. Introduction

Hydraulic handgrip dynamometers are often used to measure handgrip strength (HGS) [1]. Maximal HGS is a convenient assessment of overall muscle strength and a reliable measure of muscle function [2,3]. Muscle strength is an important characteristic of muscle function, and poor muscle function is a hallmark risk factor for unsuccessful aging [4,5]. For example, low HGS is associated with early all-cause mortality, and is a stronger predictor of all-cause and cardiovascular mortality than systolic blood pressure [6]. Thus, maximal HGS is encouraged for use in routine geriatric health assessments, and measures of HGS are becoming commonplace in clinical and research settings to determine prognosis for health conditions related to poor muscle function [7].

While HGS is a convenient assessment of strength capacity, the use of HGS has undergone criticism for being a proxy measure of overall muscle strength. For example, Yeung et al. [8] suggests that at both individual and population levels, knee extension strength and HGS showed an agreement independent of age and health status, and HGS alone should not be assumed a proxy for overall strength. Although knee extension strength may provide additional insights for muscle function, it could be considered ecologically invalid in clinical and research settings (e.g., population-based studies). Moreover, the health conditions associated with low HGS are broad, and healthcare providers that utilize HGS for assessing muscle function may experience challenges explaining what exactly low HGS is a risk factor for to their aging patients [9]. While measuring strength from the upper and lower extremities may provide a more comprehensive assessment of strength capacity [10], upper- and lower-extremity strength are related, and the information gained by collecting knee extension strength may not outweigh the feasibility lost [11]. Alternative strategies for examining muscle function should therefore diversify how muscle function is assessed, while also maintaining feasibility in measurement.

Given that HGS is solely ascertaining maximal grip force from a single hand, HGS alone could be considered unidimensional and an incomplete measure of muscle function [9]. Opportunities exist for advancing HGS protocols and how we operationalize muscle function with handgrip dynamometers by utilizing modern technologies that can include additional HGS measurements aside from maximal strength [12]. Incorporating other aspects of muscle function in HGS protocols such as digit grip strength, submaximal force control, fatigability, neuromuscular steadiness, and asymmetry will expand how muscle function is assessed, while also maintaining procedural ease and scalability. For example, HGS asymmetry and low maximal HGS together were associated with greater odds for functional disability than each measure alone [13], and HGS asymmetry is linked to early all-cause mortality [14]. As such, evaluating other aspects of muscle function outside of maximal strength may uncover deficits in muscle function that are not otherwise identified by maximal HGS. This may, in turn, improve our understanding of the underlying mechanisms of age-related motor changes for the development of screenings that help in identifying the disabling cascade. Accordingly, this pilot study sought to examine the relationships between maximal HGS, radial and ulnar digit grip strength, submaximal HGS force control, HGS fatigability, neuromuscular HGS steadiness, and HGS asymmetry in older adults. We hypothesize that the HGS variables will be differentially correlated, and that principal components would emerge from the HGS variables.

## 2. Materials and Methods

### 2.1. Participants

This study was conducted in an internal medicine unit at a clinic in Fargo, ND, USA. Individuals had to be aged at least 65 years, read and speak the English language (for consenting purposes), not have a severe cognitive impairment (also for consenting purposes), and complete HGS testing without arthritis, severe pain, or a limitation from a surgical procedure. Of the 20 individuals that completed the pre-consent screening questionnaire, 5 were unable to continue in the study because they did not meet study criteria, and 2 did not engage in data collections before study suspension due to the COVID-19 pandemic. The remaining 13 participants completed a health status questionnaire after providing written informed consent to participate in the study. Protocols were approved by the Sanford Health Institutional Review Board (STUDY00001816).

### 2.2. Handgrip Strength Measurements

All HGS measures were collected with a Biopac handgrip dynamometer and Student Lab software (Biopac Systems; Goleta, CA, USA). Previous research has suggested that Biopac handgrip dynamometers are highly reliable and valid for estimating force [15]. Four different HGS protocols were used for ascertaining HGS data: maximal HGS, radial and ulnar digit grip strength, submaximal HGS force control, and HGS fatigability. These HGS measurements took approximately 15 min to complete in total. Trained interviewers explained and demonstrated all HGS protocols before allowing participants to complete a practice trial. The hand in which HGS testing began was randomized. Participants were asked to remain seated with their shoulders adducted and neutrally rotated, elbow flexed at 90 degrees, and forearm in a neutral position. Two trials for each HGS test were collected on each hand with at least 30 s of rest between trials. Verbal encouragement was provided during trials by interviewers. Time stamps were recorded for the beginning and end of each trial because data were collected digitally with the handgrip dynamometer.

#### 2.2.1. Maximal Handgrip Strength

Participants squeezed the dynamometer with maximal effort before releasing the muscle contractions. The single greatest HGS value on either hand was included in the analyses.

#### 2.2.2. Radial and Ulnar Digit Grip Strength

Each participant squeezed the dynamometer with maximal effort, with only either their radial (digits 2 and 3) and ulnar digits (digits 4 and 5) before releasing the muscle contractions. The greatest radial and ulnar digit grip strength recorded on either hand was included in the analyses.

#### 2.2.3. Submaximal Handgrip Strength Force Control

A 25% submaximal value was calculated from the maximal HGS recorded for each hand. Interviewers asked participants to squeeze the dynamometer and maintain the 25% submaximal target grip force, as consistently as possible, for 10 s. Participants were allowed to watch the computer screen wherein data were being digitally recorded to help them maintain the 25% submaximal target as they squeezed the dynamometer. The coefficient of variation was determined over the middle 8 s time period. The best performing submaximal HGS force control value was included in the analyses [16].

#### 2.2.4. Handgrip Strength Fatigability

Participants were asked to squeeze the dynamometer at maximal effort for as long as possible. Grip force was collected starting when the dynamometer was first squeezed until the participant voluntarily released their grip on the dynamometer. A corresponding grip force curve was generated from the collected data and HGS fatigability was calculated from the fatigability index equation [17]. The lowest fatigability index, which signifies lower fatigue, on either hand was included in the analyses.

#### 2.2.5. Neuromuscular Handgrip Strength Steadiness

**Figure 1:**
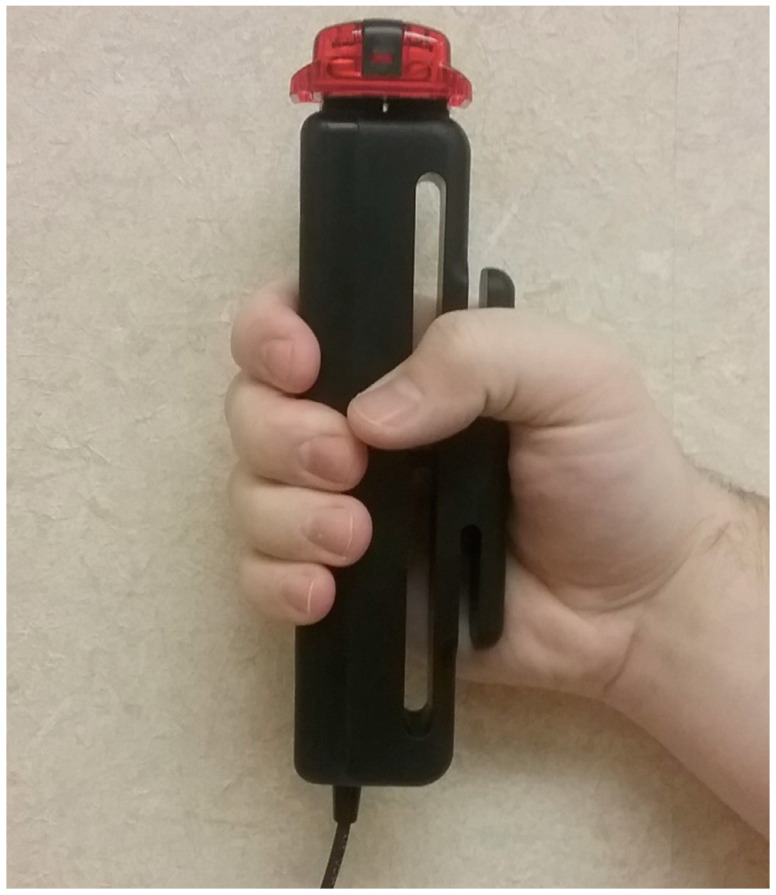
Image for how the dynamometer was securely attached to the accelerometer with velcro.

As shown in Figure 1, an ActiGraph GT3X-BT accelerometer (ActiGraph; Pensacola, FL, USA) was securely attached to the top of the handgrip dynamometer with Velcro for measuring neuromuscular HGS steadiness during all HGS tests. ActiGraph accelerometers are reliable and valid for measuring changes in body position and movement [18]. ActiLife software (ActiGraph) initialized the accelerometer at 60 Hz and processed accelerometer data. Data were stored in 1 s epochs. The specific beginning and end times of each HGS measurement were recorded to coincide with the time stamps from each HGS measure. The vector magnitudes included in the analyses were averaged for the duration of each HGS measurement that was also included.

#### 2.2.6. Handgrip Strength Asymmetry

The highest recorded HGS values on either hand were used for calculating HGS asymmetry ratio *(non-dominant HGS (kilograms)/dominant HGS (kilograms))*. Since HGS asymmetry ratios could be <1.0, any HGS asymmetry ratios <1.0 were inversed to make all HGS asymmetry ratios ≥1.0 to improve interpretability [19].

### 2.3. Statistical Analysis

All analyses were conducted with SAS 9.4 software (SAS Institute; Cary, NC, USA). The descriptive characteristics were presented as the mean ± standard deviation for continuous variables and the frequency (percentage) for categorical variables. Pearson correlations (PROC CORR) were performed to determine the relationships between maximal HGS, maximal ulnar digit grip strength, maximal radial digit grip strength, submaximal HGS force control, HGS fatigability, HGS asymmetry, maximal HGS steadiness, maximal ulnar digit grip strength steadiness, maximal radial digit grip strength steadiness, and HGS fatigability steadiness. An alpha level of 0.05 was used for all correlations.

A principal component analysis (PROC PRINCOMP) was used to derive a collection of new uncorrelated variables (i.e., principal components) for the HGS measurements that we evaluated: maximal HGS, maximal ulnar digit grip strength, maximal radial digit grip strength, submaximal HGS force control, HGS fatigability, HGS asymmetry, maximal HGS steadiness, maximal ulnar digit grip strength steadiness, maximal radial digit grip strength steadiness, and HGS fatigability steadiness. Briefly, a principal component analysis decomposes a collection of correlated variables into another collection of uncorrelated variables which are organized by descending order of variance as a form of data reduction [20,21]. Based on the Kaiser–Guttman criteria [22], principal components with eigenvalues >1.0 were retained. Specific HGS measurements with a factor loading of |>0.40| were also retained for each principal component [23]. Submaximal HGS force control neuromuscular steadiness was not included in the analyses because no tremoring was detected by the accelerometer during this measurement (i.e., the muscle contractions were not vigorous enough to generate tremoring).

## 3. Results

The descriptive characteristics of the 13 participants are presented in Table 1. Overall, participants were aged 70.9 ± 4.0 years and were mostly female (*n* = 7; 53.9%). Table 2 shows the correlations between each of the HGS measurements. Maximal HGS was positively correlated with ulnar digit strength (r = 0.91; 95% confidence interval (CI): 0.73, 0.97), radial digit strength (r = 0.94; CI: 0.82, 0.98), maximal HGS steadiness (r = 0.55; CI: 0.01, 0.84), and radial digit strength steadiness (r = 0.56; CI: 0.02, 0.85), but negatively correlated with submaximal HGS force control (r = −0.55; CI: −0.84, −0.01). Ulnar digit grip strength was positively correlated with radial digit grip strength (r = 0.93; CI: 0.80, 0.98), maximal HGS steadiness (r = 0.55; CI: 0.01, 0.84), and radial digit grip strength steadiness (r = 0.56; CI: 0.01, 0.85). Submaximal HGS force control was negatively correlated with HGS fatigability (r = −0.72; CI: −0.91, −0.29). HGS asymmetry ratio was negatively correlated with HGS fatigability steadiness (r = −0.75; CI: −0.92, −0.34). Maximal HGS steadiness was positively correlated with ulnar (r = 0.79; CI: 0.42, 0.93), and radial grip strength steadiness (r = 0.94; CI: 0.81, 0.98). Ulnar digit grip strength steadiness was also positively correlated with radial digit grip strength steadiness (r = 0.70; CI: 0.24, 0.90). Although not statistically significant, fatigability was moderately correlated with maximal strength, radial digit strength steadiness was almost largely correlated with radial digit strength, and HGS asymmetry ratio was slightly correlated with submaximal HGS force control.

**Figure 2:**
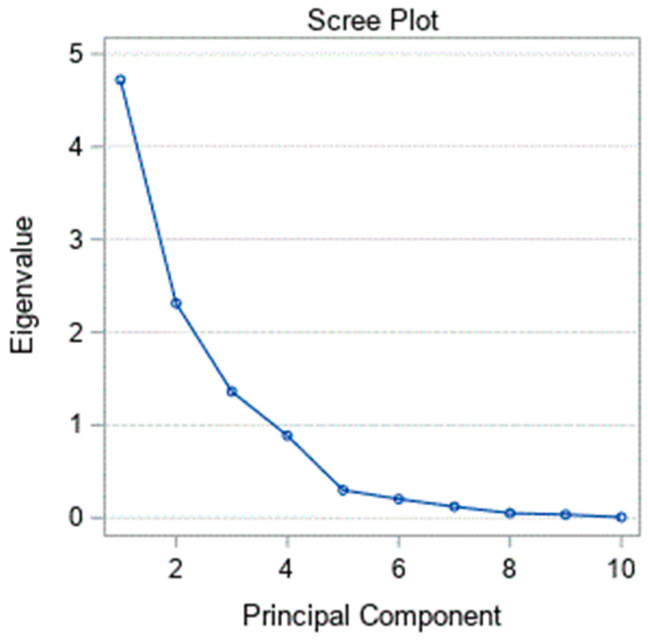
Scree plot for the principal components.

A scree plot for the principal components is shown in Figure 2. Principal components 1, 2, and 3 were retained because they had eigenvalues of 4.72, 2.31, and 1.36, respectively. The factor loadings for the principal components are revealed in Table 3. The first principal component (maximal strength) contained maximal HGS, ulnar digit grip strength, and radial digit grip strength, which explained 47.2% of the variance. The second principal component (contractile steadiness) contained maximal HGS steadiness and ulnar digit grip strength steadiness, thereby explaining 23.1% of the variance. Further, the third principal component (functional strength) contained submaximal HGS force control, HGS fatigability, HGS asymmetry, and HGS fatigability steadiness, explaining 13.6% of the variance.

## 4. Discussion

The principal results of this pilot study revealed that some of the additional HGS measurements, which may represent other aspects of muscle function, were differentially correlated. Moreover, a principal components analysis was used to maximize the variance for a linear combination of variables. Such analyses identified three dimensions for the HGS measurements that we evaluated: (1) maximal strength, (2) contractile steadiness, and (3) functional strength. These findings indicate that some of the additional HGS measurements were correlated, and other characteristics of muscle function, apart from maximal strength, could emerge from such additional HGS measurements. The identified dimensions from the principal component analysis of the HGS measurements suggest that some of these HGS measures may have separation from maximal HGS. Our research supports the potential for using modern technologies to assess muscle function, instead of hydraulic dynamometers, which could be considered archaic [12]. Although more research is needed for these additional HGS measurements, healthcare providers examining muscle function in clinical settings may consider utilizing such measures. Continued research for these HGS measures may also help us better determine if such measurements are linked to health and the failing bodily systems that could be driving age-related morbidity and disability.

Our first principal component, maximal strength, included the items maximal HGS, ulnar digit grip strength, and radial digit grip strength. Muscle contractions in a single finger generate tension in the other fingers [24]. Synchronized coordination of the digits is necessary to complete a maximal isometric grip force task such as maximal HGS [25]. When digits are not functioning uniformly, these neuromuscular deficiencies lower HGS performance [26]. While the radial digits may have greater overall grasping strength individually than the ulnar digits, the ulnar digits nonetheless contribute substantially to overall HGS [27]. Thus, it is not surprising that the HGS measurements that we evaluated, which included a maximal grip force for a short duration, were contained in a principal component.

Contractile steadiness, which was our second principal component, included the items maximal HGS steadiness and maximal ulnar digit strength steadiness. Isometric tremoring occurs when muscles contract against a rigid stationary object such as a handgrip dynamometer [28]. The ulnar nerve provides motor innervation to the majority of the intrinsic hand muscles, but particularly the ulnar digits [29]. Given that stressing the neuromuscular system with vigorous muscle contractions may lead to tremoring, this may help to explain our findings that suggest the ulnar digits could play an important role in contractile steadiness during a maximal HGS task.

The third principal component, functional strength, contained the items submaximal HGS force control, HGS fatigability, HGS asymmetry and HGS fatigability steadiness. Functional strength includes multiple muscle functions and joint activities that reflect daily living activities and functional capabilities [30]. Bimanual training has been shown to influence submaximal force control of the hands [31]. Involuntary tremoring emerges as muscles become more fatigued [32]. Wider strength asymmetries of the extremities have also been linked to greater fatigability [33]. Early fatigue may likewise influence abilities to sustain isometric force contractions [34]. The interrelationships between submaximal HGS force control, fatigability, asymmetry, and fatigability steadiness may underpin why our third principal component, functional strength, contained these items.

Some study limitations should be noted. Data collections were suspended due to the COVID-19 pandemic, which in turn, minimized our ability to recruit more study participants and meet our a priori power threshold of 50 participants. Thus, our analyses and results may have been impacted by the sample size. Assessments of reliability and validity for our HGS measurements, and continuing to refine HGS protocols, may support the use of the HGS measures we examined herein in clinical and research settings. Shifts in hand lateralization could not be accounted for in our study design. Hand dominance was self-reported, and all of our participants were recorded as right hand dominant. Therefore, the generalizability for our findings was limited to those who are left handed. Future studies with higher sample sizes, longitudinal study designs, and heterogeneity in participant descriptive characteristics may help to support or dispute our findings.

## 5. Conclusions

This pilot study found that additional HGS measurements, as measured with a digital handgrip dynamometer and accelerometer, may reveal other aspects of muscle function that are not otherwise identified by maximal HGS alone. Our findings suggest that maximal HGS could be an incomplete measure of muscle function, and that including additional HGS measurements that better resemble muscle function could improve how we assess muscle function for age-related health conditions. Future research is warranted for evaluating how maximal strength, contractile steadiness, and functional strength may factor into the prognostic utility of HGS assessments, including the use of a potential battery from these factors. Such research would diversify HGS protocols while preserving their feasibility, and clinical and research value.

## Figures and Tables

**Table 1:** Descriptive Characteristics of the Participants.

Variables	*n* = 13
Age (years)	70.9 ± 4.0
Female (*n* (%))	7 (53.9)
Height (centimeters)	171.0 ± 10.4
Body Mass (kilograms)	81.1 ± 21.0
Body Mass Index (kilograms per meters-squared)	27.4 ± 4.8
Married (*n* (%))	10 (76.9)
Retired (*n* (%))	9 (69.2)
Completed College (*n* (%))	9 (69.2)
Right Hand Dominant (*n* (%))	13 (100.0)
Self-Rated Health (*n* (%))	
Excellent or Very Good	6 (46.2)
Good, Fair, or Poor	7 (53.8)
Maximal Handgrip Strength (kilograms)	22.6 ± 7.4
Maximal Ulnar Digit Strength (kilograms)	8.0 ± 2.5
Maximal Radial Digit Strength (kilograms)	13.1 ± 4.1
Submaximal Handgrip Strength Force Control (coefficient of variation)	21.9 ± 4.2
Handgrip Strength Fatigability Index	16.4 ± 7.1
Handgrip Strength Asymmetry Ratio	1.1 ± 0.1
Maximal HGS Steadiness (vector magnitude)	7.5 ± 13.7
Maximal Ulnar Digit Strength Steadiness (vector magnitude)	12.0 ± 16.1
Maximal Radial Digit Strength Steadiness (vector magnitude)	8.2 ± 16.3
Handgrip Strength Fatigability Steadiness (vector magnitude)	0.4 ± 0.5

Note: results are presented as the mean ± standard deviation or the frequency (percentage) where indicated.

**Table 2:** Correlations between the handgrip strength measurements.

Variables	Maximal Strength	Ulnar Digit Strength	Radial Digit Strength	Submaximal Force Control	Fatigability	Asymmetry
Maximal Strength	-	-	-	-	-	-
Ulnar Digit Strength	r = 0.91; *p* < 0.01	-	-	-	-	-
Radial Digit Strength	r = 0.94; *p* < 0.01	r = 0.93; *p* < 0.01	-	-	-	-
Submaximal Force Control	r = −0.55; *p* = 0.04	r = −0.45; *p* = 0.12	r = −0.50; *p* = 0.07	-	-	-
Fatigability	r = 0.43; *p* = 0.13	r = 0.19; *p* = 0.51	r = 0.28; *p* = 0.35	r = −0.72; *p* < 0.01	-	-
Asymmetry	r = 0.43; *p* = 0.13	r = 0.44; *p* = 0.12	r = 0.29; *p* = 0.32	r = −0.21; *p* = 0.48	r = 0.07; *p* = 0.80	-
Maximal Steadiness	r = 0.55; *p* = 0.04	r = 0.55; *p* = 0.04	r = 0.52; *p* = 0.06	r = −0.10; *p* = 0.72	r = 0.15; *p* = 0.61	r = −0.10; *p* = 0.72
Ulnar Digit Strength Steadiness	r = 0.26; *p* = 0.37	r = 0.26; *p* = 0.38	r = 0.25; *p* = 0.39	r = 0.19; *p* = 0.51	r = −0.06; *p* = 0.83	r = −0.15; *p* = 0.61
Radial Digit Strength Steadiness	r = 0.56; *p* = 0.04	r = 0.56; *p* = 0.04	r = 0.48; *p* = 0.09	r = −0.13; *p* = 0.66	r = 0.17; *p* = 0.56	r = −0.01; *p* = 0.98
Fatigability Steadiness	r = −0.51; *p* = 0.07	r = −0.41; *p* = 0.16	r = −0.32; *p* = 0.27	r = 0.24; *p* = 0.42	r = −0.32; *p* = 0.28	r = −0.75; *p* < 0.01
**Variables**	**Maximal Strength Steadiness**	**Ulnar Digit Strength Steadiness**		**Radial Digit Strength Steadiness**		**Fatigability Steadiness**
Ulnar Digit Strength Steadiness Radial Digit Strength Steadiness Fatigability Steadiness	r = 0.79; *p* < 0.01	**-**		**-**		**-**
	r = 0.94; *p* < 0.01	r = 0.70; *p* < 0.01		**-**		**-**
	r = −0.13; *p* = 0.67	r = −0.13; *p* = 0.66		r = −0.15; *p* = 0.60		**-**

Note: r-values are the correlation coefficients.

**Table 3:** Factor loadings for the principal component analysis of the handgrip strength measures.

Variables	Principal Component 1	Principal Component 2	Principal Component 3
Maximal Handgrip Strength	0.44 *	−0.11	−0.01
Maximal Ulnar Digit Strength	0.42 *	−0.05	0.10
Maximal Radial Digit Strength	0.41 *	−0.05	−0.03
Submaximal Handgrip Strength Force Control	−0.25	0.34	0.48
Handgrip Strength Fatigability	0.20	−0.26	−0.52 *
Handgrip Strength Asymmetry	0.18	−0.39	0.53 *
Maximal Handgrip Strength Steadiness	0.33	0.42 *	−0.06
Maximal Ulnar Digit Strength Steadiness	0.21	0.49 *	0.14
Maximal Radial Digit Strength Steadiness	0.34	0.38	−0.03
Handgrip Strength Fatigability Steadiness	−0.25	0.28	−0.42 *
Variance Explained	47.2%	23.1%	13.6%

Note: * significant factor loading (|>0.40|).
